# Predictions of Hot Spot Residues at Protein-Protein Interfaces Using Support Vector Machines

**DOI:** 10.1371/journal.pone.0016774

**Published:** 2011-02-28

**Authors:** Stefano Lise, Daniel Buchan, Massimiliano Pontil, David T. Jones

**Affiliations:** Department of Computer Science, University College London, London, United Kingdom; Massachusetts Institute of Technology, United States of America

## Abstract

Protein-protein interactions are critically dependent on just a few ‘hot spot’ residues at the interface. Hot spots make a dominant contribution to the free energy of binding and they can disrupt the interaction if mutated to alanine. Here, we present HSPred, a support vector machine(SVM)-based method to predict hot spot residues, given the structure of a complex. HSPred represents an improvement over a previously described approach (Lise *et al*, BMC Bioinformatics 2009, **10**:365). It achieves higher accuracy by treating separately predictions involving either an arginine or a glutamic acid residue. These are the amino acid types on which the original model did not perform well. We have therefore developed two additional SVM classifiers, specifically optimised for these cases. HSPred reaches an overall precision and recall respectively of 61% and 69%, which roughly corresponds to a 10% improvement. An implementation of the described method is available as a web server at http://bioinf.cs.ucl.ac.uk/hspred. It is free to non-commercial users.

## Introduction

Alanine scanning mutagenesis is a powerful experimental methodology for investigating the structural and energetic characteristics of protein complexes [1]. Individual amino-acids are systematically mutated to alanine and changes in free energy of binding (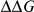) measured. As alanine amino acids do not have a side-chain beyond the 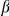-carbon, this procedure in effect tests the importance of individual side-chain groups for complex formation, providing a map of the so-called functional epitope. Results from a number of experiments indicate that only a small subset of contact residues contribute significantly to the binding free energy. These residues have been termed ‘hot spots’ and if mutated they can disrupt the interaction. For the majority of interface residues instead, the effect of an alanine mutation is minimal [2].

Hot spots are typically defined as those residues for which 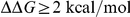. In recent years, several computational approaches have been developed to identify them at protein-protein interfaces [3]–[16]. Accurate predictive models provide a valuable complement to experimental studies and add to our understanding of the factors that influence affinity and specificity in protein-protein interfaces. In addition, they can have important applications in the field of drug discovery. A number of recent studies have been successful in developing (drug-like) small molecules that bind at hot spots and inhibit complex formation [17]. Reliable hot spots predictions could therefore represent the first step in rational drug design projects [18].

In a previous work, we presented a machine learning strategy to identify hot spot residues in protein-protein interfaces, given the structure of the complex [12]. We considered the basic energetic terms that contribute to hot spot interactions, i.e. van der Waals potentials, solvation energy, hydrogen bonds and Coulomb electrostatics, and treated them as input features of a Support Vector Machine (SVM) classifier. We found that the method could predict hot spots with overall good accuracy, comparing favourably to other available approaches. However, by grouping mutations according to the amino acid type, we observed that in some cases the SVM model did not perform too well, for example on predictions involving arginine or glutamic acid residues.

In this paper, we report the development of HSPred, a hot spot prediction method that aims to overcome the limitations highlighted above. For this purpose, we have integrated the original approach with two additional SVM classifiers, specifically built for mutations involving Arg and Glu residues. The two additional models are trained on the same data set as the ‘general’ model but are biased to perform well on Arg and Glu due to a different choice of input features. Employing a strict cross-validation scheme, we show that this strategy leads to a significant improvement over the previous version of the method. We further validate the results by applying HSPred to an external test case, which is not part of the original data set.

## Results and Discussion

**Figure 1:**
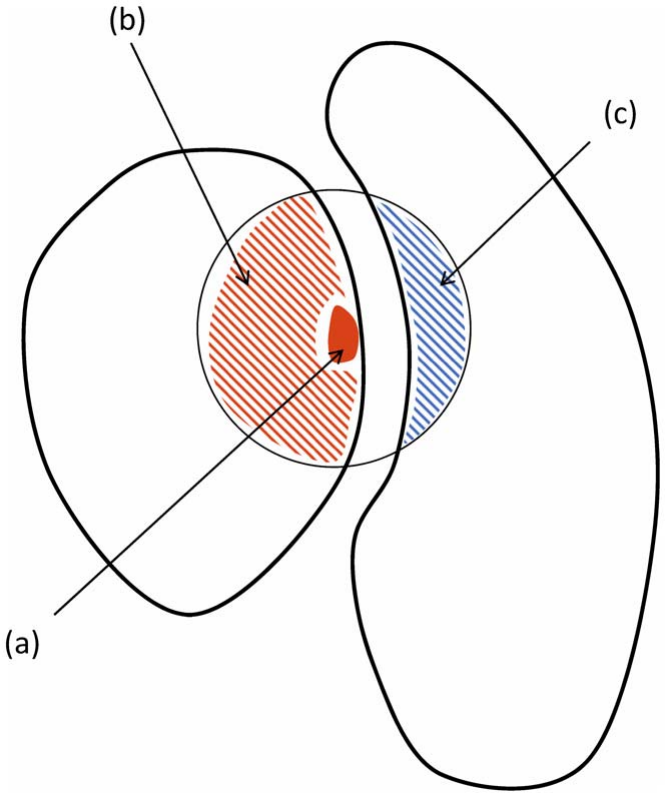
Schematic overview of protein structural regions which define the different energy contributions. The red filled area, (a), corresponds to side-chain atoms of the mutated residue; the red and blue striped regions, (b) and (c) respectively, correspond to atoms within 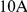 of the 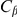 of the mutated residue. We distinguish 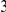 types of interactions: *side-chain inter-molecular* between (a) and (c), *environment inter-molecular* between (b) and (c), *side-chain intra-molecular* between (a) and (b).

The problem we have investigated is the prediction of hot spot residues at a protein-protein interface using a machine learning approach. As input variables, we have considered basic energy terms (van der Waals, hydrogen bond, electrostatic and desolvation potentials) calculated from the complex structure. We have distinguished contributions from different structural regions in the complex, leading to 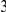 distinct types of interactions: side-chain inter-molecular, environment inter-molecular and side-chain intra-molecular (see Figure 1). To each of them, we have associated 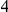 input features, corresponding to the energy terms above. In total therefore there are 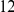 input features but some of them have not been included in our models because scarcely informative (see Materials and Methods for more details). Support Vector Machines (SVMs) have then be used to learn from a training set to classify residues as hot spots 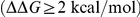 or non hot spots 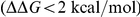.

We have built a classifier, SVM_X_, based on the following 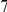 features: van der Waals, hydrogen bond and solvation side-chain inter-molecular energies; van der Waals, hydrogen bond and solvation environment inter-molecular energies; van der Waals side-chain intra-molecular energy. A summary of the results is reported in Table 1 according to various performance measures. The precision 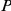 is the fraction of true hot spots among the set of residues predicted to be hot spots; the recall 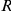 is the fraction of correctly identified hot spots relative to all those present in the data set; the 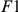 score is a weighted average of the precision and recall; the Matthews Correlation Coefficient (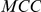) is a commonly used measure of the quality of binary classifications (see Methods section for more details). SVM_X_ is very similar in its design and performance to the model described in [12]. With respect to the latter, SVM_X_ does not rely on any electrostatic term but it includes the van der Waals side-chain intra-molecular energy. We report in Table 2 the weight of each energy term in the linear scoring function.

**Table 1:** Summary of results.

Model	Precision	Recall	F1 score	MCC
SVM 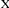	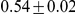	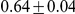	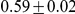	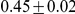
HSPred	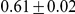	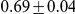	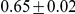	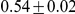

Cross-validated estimates of performances for SVM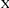 and HSPred. MCC is the Matthews correlation coefficient (see Methods section for definition of the various performance measures).

**Table 2:** Weight of energy terms in the scoring functions.

Feature (energy term)	SVM 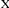	SVM 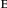	SVM 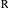
Side-chain inter-molecular			
van der Waals	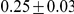	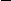	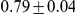
hydrogen bond	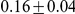	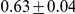	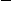
electrostatics	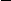	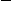	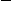
desolvation	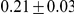	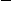	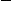
Environment inter-molecular			
van der Waals	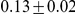	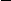	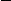
hydrogen bond	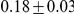	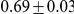	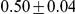
electrostatics	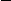	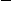	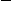
desolvation	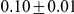	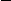	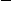
Side-chain intra-molecular			
van der Waals	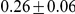	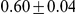	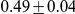
hydrogen bond	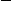	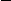	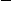
electrostatics	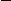	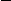	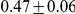
desolvation	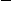	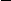	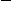
Threshold	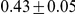	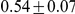	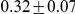

We report the absolute value of the weight associated to each feature in the scoring functions, together with the threshold that defines the decision boundary. Energy terms which are not included in the scoring function are denoted with the 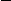 symbol.

**Figure 2:**
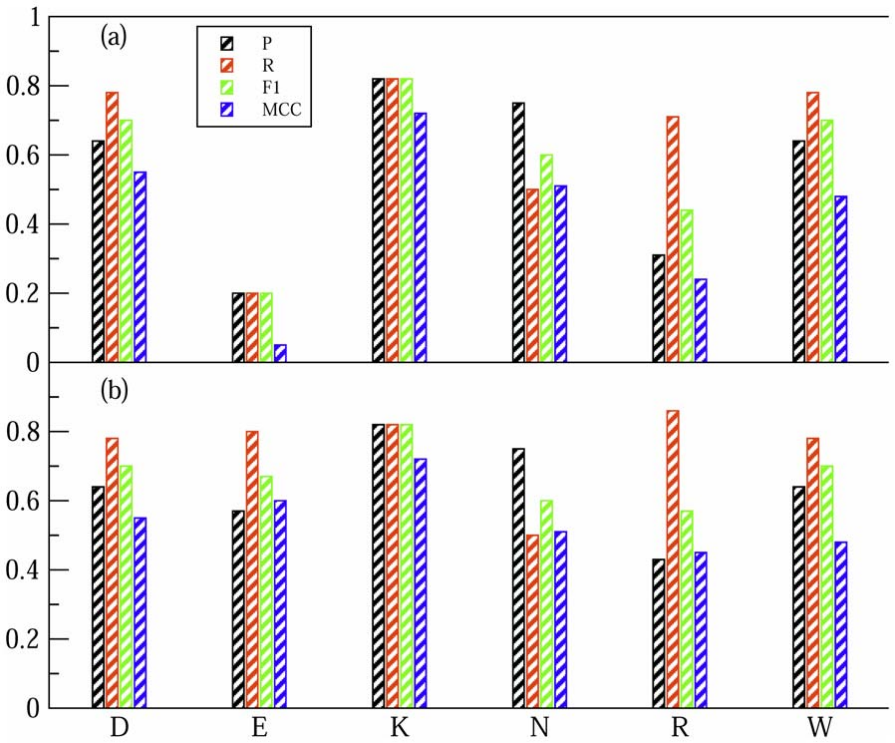
Predictions results for different amino acids. Only the most frequent amino acid in the database are reported. In (a) are the results for SVM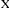, in (b) for HSPred, which includes SVM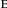 and SVM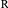.

We have analysed the SVM_X_ predictions by grouping mutations according to the amino acid type. In Figure 2(a) we report the results for the most frequent amino acids in the database. SVM_X_ has a good accuracy over most of amino acid types and is not biased toward some specific amino acid property (e.g. hydrophobic or charged residues). At the same time, however, it does not perform so well on mutations involving Arg and Glu. To tackle this problem, we have developed two additional classifiers, respectively SVM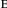 and SVM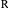, specifically optimised for these amino acids. SVM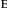 and SVM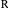 have been trained using the whole data set but differ from SVM_X_ for the choice of input features and the associated weights (see Table 2).

As can be seen in Figure 2(b), SVM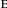 and SVM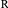 achieve significantly improved results on Glu and Arg predictions. A further confirmation of the improvement comes from analysing the correlation coefficients 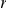 between the classifiers output scores and the observed 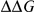 values. For Glu residues, 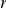 increases from 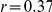 for SVM_X_ to 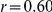 for SVM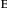; for Arg, 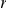 increases from 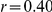 for SVM_X_ to 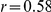 for SVM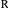. This suggests that SVM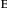 and SVM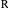 are indeed more effective than SVM_X_ in describing mutations involving Glu and Arg residues, respectively, and that the observed improvement is genuine and not due to over-fitting.

We have combined SVM_X_, SVM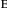 and SVM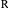 into a unique classifier, HSPred. SVM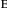 and SVM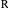 act respectively on Glu and Arg amino acids, SVM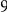 on all other amino acid types. We report a summary of the results for HSPred in Table 1. HSPred performs significantly better than SVM_X_, reflecting the inclusion of SVM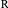 and SVM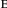. As can be seen from Figure 2(b), predictions on Arg and Glu are roughly as accurate as for the other residues. HSPred therefore successfully overcomes the major limitations of the previously proposed method [12]. Most notable is the improvement on Glu predictions.

**Figure 3:**
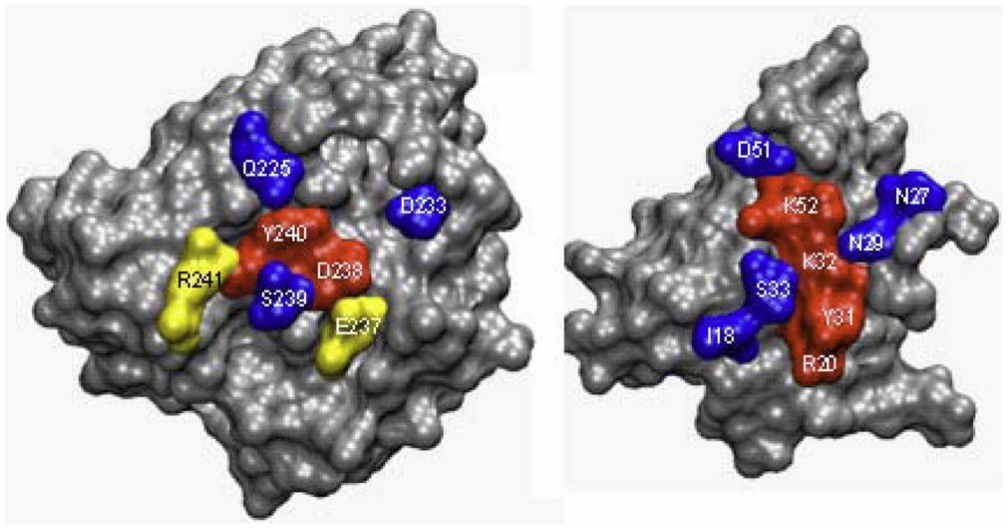
Ras/RalGDS complex. Mapping of HSPred predictions onto the the complex (PDB code: 1LFD). The monomers have been rotated to display the interface. Red residues are correctly predicted hot spots (true positives); blue residues are correctly predicted non hot spots (true negatives); yellow residues are non hot spots erroneously predicts as hot spots (false positives).

To further validate HSPred, we have applied it to the protein-protein complex Ras/RalGDS (PDB code: 1LFD). The Ras/RalGDS complex is not homologous to any of the complexes in the original data set and it can then be regarded as an independent external test case. Experimental 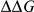 values are available in [19], from which we have taken the data corresponding to 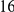 interface alanine mutations (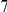 on Ras and 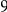 on RalGDS). HSPred correctly identifies 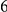 hot spot (true positives) and 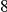 non hot spot residues (true negatives). However, 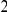 residues are wrongly predicted as hot spots (false positives). The predictions are illustrated in Figure 3. These results are in line with the cross-validated estimates in Table 1 and confirm the accuracy of HSPred.

**Figure 4:**
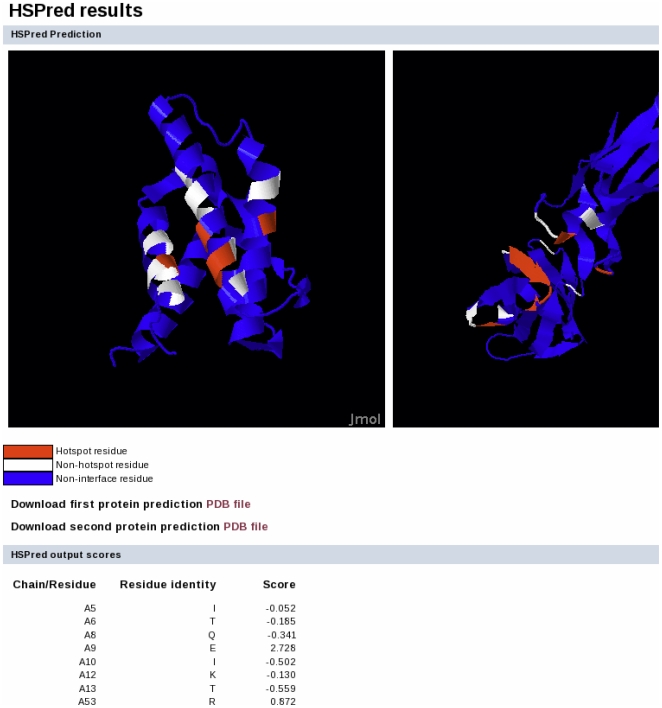
Sample output for the HSPred server. Screenshot of the results page for the IL-4/IL-4R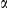 complex (PDB code: 1IAR). On top, predictions are visualised using a Jmol applet. On the left is IL-4 (chain A), on the right IL-4R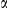 (chain B). Predicted hot spots are in red, non hot spots in white. Residues not part of the interface are in blue. Below, predictions scores for each interface residues (excluding Pro and Gly amino acids) are reported (note that only the first few residues are displayed here). Scores greater than zero corresponds to predicted hot spots.

We have implemented HSPred as a fully automatic web server, available at http://bioinf.cs.ucl.ac.uk/hspred. As input it requires a PDB formatted file containing the structure of the protein-protein complex. The user needs to define the interface to analyse by specifying the chain identifiers for each protein on either side of the interface. The output consists of two components: (i) a Jmol applet to visualise and explore the predictions using the protein structures and (ii) a table listing HSPred scores for each interface amino acid. The output page for an illustrative example is reported in Figure 4. The complex tested is Interleukin 4 (IL-4) bound to its receptor 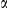 chain (IL-4R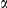) (PDB code: 1IAR). Alanine mutational data from experiments are available for this complex [20], [21]. Out of 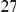 interface mutations, HSPred predicts 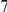 true positives, 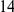 true negatives, 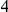 false positive and 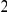 false negatives. These results further validate the predictive accuracy of HSPred.

To conclude, in this paper we have described HSPred, an accurate and reliable computational method to predict hot spot residues at protein-protein interfaces, given the structure of a complex. HSPred is available as a web server and it is free for non-commercial users. We believe that HSPred predictions will be useful in guiding biomedical experiments. In particular, we are currently testing its capacity to identify druggable binding sites at protein-protein interfaces [22].

## Materials and Methods

### Data sets

In our study, we have used the same data set as in [12]. It consists of 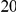 protein complex structures for which alanine mutational data are available. Only protein-protein interactions involving an extended interface are included (we have therefore ignored protein-peptide complexes). Following previous publications [23], we define hot spots as those alanine mutations for which 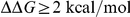 (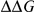 is the change in binding free energy). Only mutations occurring at the complex interface are retained. In total the data set comprises 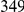 mutations, of which 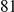 correspond to hot spots. For cross-validation purposes, we have grouped homologous complexes and formed 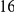 non-homologous clusters. Accordingly, we have implemented a 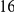-fold cross-validation strategy. A detailed description of the data set, individual mutations and clustering criteria can be found in [12].

In addition, we have applied HSPred to the Ras/RalGDS protein-protein complex (PDB code: 1LFD) for which experimental 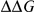 values are available [19]. From the original reference, we have taken the data corresponding to 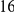 interface alanine mutations. As the Ras/RalGDS complex is not homologous to any of the complexes in the original data set, it can be regarded as an independent external test case. A similar data set had been used previously in [13] for validation purposes.

As a further illustrative example we have applied HSPred to Interleukin-4 (IL-4) bound to its receptor 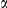 chain (IL-4R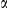) (PDB code: 1IAR). Experimental 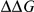 values are available for this complex too [20], [21]. The IL-4/IL-4R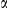 complex is likely a remote homologue of the complex between human growth hormone (hGH) and its binding protein (hGHbp), which is part of our training set (PDB code: 1A22). IL-4 and hGH share only 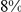 sequence identity by optimal structural alignment but belong to the same homologous superfamily group (H-level) according to the CATH database [24]. Similarly, the sequence identity between IL-4R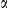 and hGHbp is only 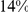 but structural similarity suggests a homology relationship. It has however been pointed out that the IL-4/IL-4R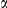 complex differs in several important functional and structural aspects from the hGH/hGHbp complex [20], [21], [25]. It could therefore in effect be regarded as an additional independent test case.

### Input features

As input features for the Support Vector Machines we have used basic energy terms that have been found to be important for the stability of protein complexes. These are van der Waals potential, hydrogen bonds, Coulomb electrostatics and desolvation energy. We distinguish contributions from 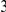 different structural regions (schematised in Figure 1):

- *Side-chain inter-molecular energies*: interaction energies between side-chain atoms of the mutated residue and atoms in the partner protein (respectively atoms in the red filled area and blue striped area in Figure 1).
- *Environment inter-molecular energies*: interaction energies between atoms in the two proteins that are within 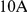 of the 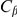 of the mutated residue (respectively atoms in the red striped area and blue striped area in Figure 1). We do not include the contribution from the mutated side-chain in this term.
- *Side-chain intra-molecular energies*: interaction energies between side-chain atoms of the mutated residue and other atoms in the same protein (respectively atoms in the red filled area and red striped area in Figure 1).

In total therefore there are 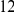 input features (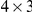), although not all of them have been used to build our SVM models (we discuss our feature selection below). A detailed description of how energy components are calculated from the PDB structures is reported in [12].

### Support Vector Machines models

We have used the program package SVM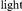 [26], which is available at the website http://svmlight.joachims.org/. As in [12], we have opted for a linear kernel and implemented a nested-loop cross-validation scheme. The latter consists of two nested cross-validation loops: an outer one for testing, an inner one for choosing hyper-parameters. In the inner cycle, the hyper-parameters are optimised by applying a grid search and the model performance is assessed by means of the F1 score. The nested-loop cross-validation scheme allows also to estimate statistical errors on performance measures (see [12] for details).

#### Models construction and feature selection

We have analysed the correlation coefficients 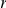 between energy features and the observed 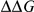 values (see Table 3). We have then built a ‘baseline’ model, SVM_X_, including only the 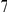 features for which 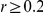. These are: van der Waals, hydrogen bond and solvation side-chain inter-molecular energies, van der Waals, hydrogen bond and solvation environment inter-molecular energies, and van der Waals side-chain intra-molecular energy. Note that the values of the correlation coefficients do not vary sensibly in the 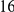 different training sets, implying that this choice of features is robust.

**Table 3:** Correlation of energy terms with observed 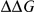 values.

Feature (energy term)	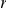
Side-chain inter-molecular	
van der Waals	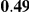
hydrogen bond	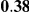
electrostatics	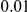
desolvation	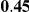
Environment inter-molecular	
van der Waals	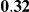
hydrogen bond	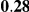
electrostatics	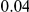
desolvation	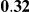
Side-chain intra-molecular	
van der Waals	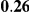
hydrogen bond	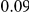
electrostatics	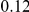
desolvation	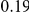

We report the absolute values of the correlation coefficients 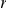 between energy features and the observed 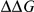 (values greater than 0.2 are in bold).

We have analysed the predictions of SVM_X_ by grouping mutations according to the amino acid type. In particular we have focused on the most frequent amino acids in our data set, i.e. those occurring more than 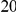 times with at least 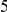 hot spot examples. The list comprises the following 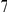 amino acid types: Arg, Asn, Asp, Glu, Lys, Trp and Tyr. We observe a good performance for all amino acids except Arg and Glu for which 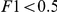 (see Figure 2). To overcome these limitations, we have built two separate SVM classifiers, SVM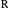 and SVM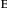, for mutations involving respectively Arg and Glu.

In theory, one could use the amino acid identity as input feature or build a model using only, e.g., Glu mutations. In practice, at present this is not feasible as there are not enough mutational data. We have reasoned instead that SVM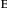 and SVM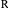 should not be completely different from SVM_X_, rather they should differ only marginally from the latter. In this spirit, we have trained several different but related models. All models are trained using the whole data set (comprising therefore mutations from all amino acid types) but each of them corresponds to a different choice of input features. Within this ensemble of classifiers we have selected those that best perform on Arg and Glu.

Our strategy has been to bias the classifiers to perform well on Arg and Glu by selecting a specific subset of features. This reflects the observation that some energy features appear to be more important for some amino acids than for others, i.e. for some amino acid they correlate better with the observed 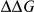
s. Note that the hyper-parameters in each of the models in the ensemble are optimised over all the mutations in the training set. The identity of the amino acid of interest enters only when selecting the best model within the ensemble. We find this to be a robust strategy, i.e. it is not too sensitive to small modifications in the training set.

Given the starting 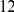 features, there is a huge number of possible combinations that can be selected and it is clearly not feasible to test them all. To simplify the problem, we have considered only combinations with 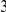 or 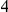 features, taken from the 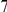 features used for SVM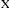. We have further constrained the selection by excluding pairs of highly correlated features, i.e. features for which 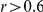, because they would be redundant. For example, only one term between the van der Waals and solvation side-chain inter-molecular energies can be included. Similarly only one term among the 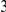 environment energies can be chosen. With these constraints, there are a total of 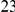 different feature combinations (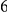 combinations having 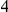 features and 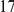 having 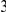 features). We have built a classifier for each of them and then selected the one performing best on, e.g., Glu. In the case of Arg, the intra-molecular coulomb term appears to be also important (correlation coefficient with observed 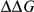 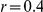). We have therefore tested additional 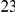 combinations which are obtained by adding the intra-molecular coulomb term to the set above.

It is important to underline that when assessing the results for SVM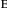 and SVM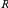 by cross-validation, the choice of the best model (feature combination) is performed within the inner loop of the nested-loop cross-validation scheme (i.e. using the training set only), similarly to the choice of hyper-parameters. This ensures that the optimal feature combination for either Arg or Glu is selected without ever considering the performance on the test set. It is worth noting that for both Arg and Glu the feature combination that gives the best results is consistent in the 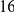 different training sets. For example for Glu the optimal feature combination is always hydrogen bond side-chain inter-molecular, hydrogen bond environment and van der Waals side-chain intra-molecular. It is also worth mentioning that Glu and Arg can be singled out based on the performance of SVM_X_ in the training sets, therefore complying to the cross-validation scheme. We have not explicitly stated it above to keep the discussion as simple as possible.

### Measures of prediction performance

We primarily assess the prediction performances of our method using the F1 score. Let 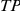, 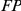, 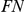 refer to the number of true positives, false positives and false negative respectively. Precision (P, also called specificity) and recall (R, also called sensitivity) are defined as 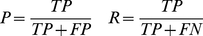
(1)


The F1 score is the harmonic mean of precision and recall 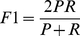
(2)


We also calculate the Matthew's correlation coefficient (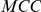) given by 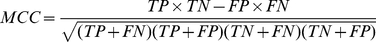
(3)


where 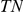 is the number of true negative and 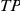, 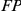 and 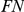 are as above.
